# Targeted Oligonucleotides for Treating Neurodegenerative Tandem Repeat Diseases

**DOI:** 10.1007/s13311-019-00712-9

**Published:** 2019-05-16

**Authors:** Rula Zain, C. I. Edvard Smith

**Affiliations:** 1 Department of Laboratory Medicine, Centre for Advanced Therapies, Karolinska Institutet, Karolinska University Hospital, SE-141 86 Stockholm, Sweden; 2 Department of Clinical Genetics, Centre for Rare Diseases, Karolinska University Hospital, SE-171 76 Stockholm, Sweden

**Keywords:** Chromatin, DNA repair, fragile X syndrome, locked nucleic acid, spinal and bulbar muscular atrophy, non-canonical DNA structure

## Abstract

Nucleotide repeat disorders encompass more than 30 diseases, most of which show dominant inheritance, such as Huntington’s disease, spinocerebellar ataxias, and myotonic dystrophies. Yet others, including Friedreich’s ataxia, are recessively inherited. A common feature is the presence of a DNA tandem repeat in the disease-associated gene and the propensity of the repeats to expand in germ and in somatic cells, with ensuing neurological and frequently also neuromuscular defects. Repeat expansion is the most frequent event in these diseases; however, sequence contractions, deletions, and mutations have also been reported. Nucleotide repeat sequences are predisposed to adopt non-B-DNA conformations, such as hairpins, cruciform, and intramolecular triple-helix structures (triplexes), also known as H-DNA. For gain-of-function disorders, oligonucleotides can be used to target either transcripts or duplex DNA and in diseases with recessive inheritance oligonucleotides may be used to alter repressive DNA or RNA conformations. Most current treatment strategies are aimed at altering transcript levels, but therapies directed against DNA are also emerging, and novel strategies targeting DNA, instead of RNA, are described. Different mechanisms using modified oligonucleotides are discussed along with the structural aspects of repeat sequences, which can influence binding modes and efficiencies.

**Electronic supplementary material:**

The online version of this article (10.1007/s13311-019-00712-9) contains supplementary material, which is available to authorized users.

## Introduction

**Fig. 1:**
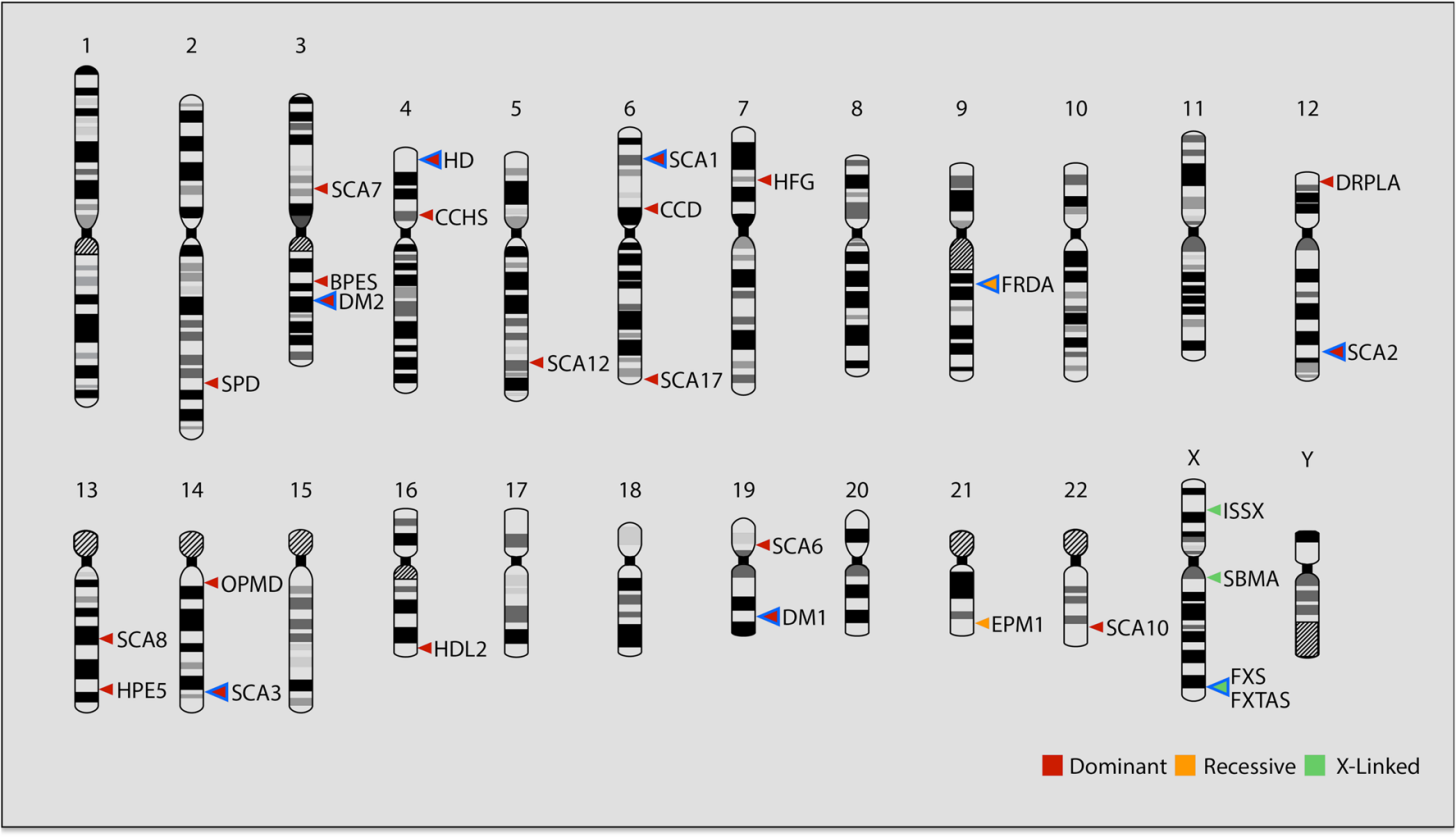
Chromosomal location of genes in nucleotide repeat disorders. BPES (blepharophimosis, ptosis, and epicanthus inversus syndrome), gene: *FOXL2*, forkhead box L2; CCD (cleidocranial dysplasia), gene: *RUNX2*, runt-related transcription factor 2; CCHS (congenital central hypoventilation syndrome), gene: *PHOX2B*, paired like homeobox 2B; DM1 (myotonic dystrophy type 1), gene: *DMPK*, dystrophia myotonica protein kinase; DM2 (myotonic dystrophy type 2), gene: *CNBP*, CCHC-type zinc finger nucleic acid–binding protein; DRPLA (dentatorubral-pallidoluysian atrophy), gene: *ATN1*, atrophin 1; EPM1 (progressive myoclonus epilepsy), gene: *CSTB*, cystatin B; FRDA (Friedreich’s ataxia), gene: *FXN*, frataxin; FXS (fragile X syndrome), gene: *FMR1*, fragile X mental retardation 1; FXTAS (fragile X-associated tremor ataxia syndrome), *FMR1*, fragile X mental retardation 1; HD (Huntington’s disease), gene: *HTT*, huntingtin; HDL2 (Huntington’s disease–like 2), gene: *JPH3*, junctophilin 3; HFG (hand-foot-genital-syndrome), gene: *HOXA13*, homeobox A13; HPE5 (holoprosencephaly 5), gene: *ZIC2*, zinc finger protein of cerebellum 2; ISSX (X-linked infantile spasms), gene: *ARX*, aristaless-related homeobox; OPMD (oculopharyngeal muscular dystrophy), gene: *PABPN1*, poly(A) binding protein nuclear 1; SBMA (spinal and bulbar muscular atrophy), gene: *AR*, androgen receptor; SCA1 (spinocerebellar ataxia type 1), gene: *ATXN1*, ataxin 1; SCA2 (spinocerebellar ataxia type 2), gene: *ATXN2*, ataxin 2; SCA3 (spinocerebellar ataxia type 3), gene: *ATXN3*, ataxin 3; SCA6 (spinocerebellar ataxia type 6), gene: *CACNA1A*, calcium voltage–gated channel subunit alpha1 A; SCA7 (spinocerebellar ataxia type 7), gene: *ATXN7*, ataxin 7; SCA8 (spinocerebellar ataxia type 8), gene: *ATXN8OS*, ATXN8 opposite strand lncRNA; SCA10 (spinocerebellar ataxia type 10), gene: *ATXN10*, ataxin 10; SCA12 (spinocerebellar ataxia type 12), gene: *PPP2R2B*, protein phosphatase 2 regulatory subunit B beta; SCA17 (spinocerebellar ataxia type 17), gene: *TBP*, TATA-box–binding protein; SPD (synpolydactyly 1), gene: *HOXD13*, homeobox D13. Red arrows indicate dominant inheritance, orange autosomal recessive, and green X-linked inheritance. Arrows surrounded by blue mark diseases described in some detail in the review.

Nucleotide repeat disorders (NRDs) are defined by the presence of tandem copies of a specific DNA sequence within the disease-associated gene(s) [1, 2]. DNA repeat sequences are located throughout the genome on both autosomes and on the X chromosome, as depicted in Fig. 1, and pathologic repeat expansion may occur on one or both alleles. The number of inherited repeats varies between the different disease genes and among individuals within the same patient group. Even if premutations with an increased number of repeats are inherited, further expansion still occurs in somatic cells, and another critical feature is that the expansion is tissue-specific, suggesting that phenotypic differences among cell types may determine the repeat instability [3–5]. The most studied are trinucleotide repeat (TNRs) sequences; however, larger repeats also exist in the human genome, such as tetra-, penta-, and hexanucleotide repeats. Expanded repeat sequences are found in both coding and non-coding gene regions, and some examples are shown in Table 1. A common denominator of nucleotide repeats is their inclination to expand, which results in the introduction of a varying number of sequence copies and consequently mutation of the corresponding gene. The genomic instability component in NRDs presents an additional dimension as compared to genetic diseases carrying variations in non-repeat sequences. The number of expanded repeats, in most cases, is directly correlated with age at onset and severity of disease, and therefore, expansion in one specific gene can result in varying subphenotypes within the same disorder. Oligonucleotide (ON)-targeting strategies have recently proved to be successful for the treatment of an increasing number of genetic diseases [6]. Nevertheless, the medical research field of ON treatment in NRDs is still at its infancy.

**Table 1:** Nucleotide repeat disorders: disease gene characteristics

Disease (abbreviation)	Gene	Normal repeat length	Expanded repeat length	Gene product	Repeat sequence	Location of repeat
Fragile X mental retardation 1, (Fragile X)	*FMR1*	5–55	> 200	Fragile X mental retardation protein	CGG•CCG	5′ UTR
Friedreich’s ataxia (FRDA)	*FXN*	5–34	66–1700	Frataxin	GAA•CTT	Intron
Huntington’s disease (HD)	*HTT*	6–35	36–250	Huntingtin	CAG•CTG	Exon
Myotonic dystrophy type 1 (DM1)	*DMPK*	5–34	> 50	Dystrophia myotonica protein kinase	CTG•CAG	3′ UTR
Myotonic dystrophy type 2 (DM2)	*CNBP*	11–26	75–11,000	Cellular nucleic acid–binding protein	CCTG•CAGG	Intron
Spinal and bulbar muscular atrophy (SBMA)	*AR*	9–34	38–68	Androgen receptor	CAG•CTG	Exon
Spinal cerebellar ataxia type 1 (SCA1)	*ATXN1*	6–44	39–82	Ataxin 1	CAG•CTG	Exon
Spinal cerebellar ataxia type 2 (SCA2)	*ATXN2*	12–44	55–87	Ataxin 2	CAG•CTG	Exon
Spinal cerebellar ataxia type 3 (SCA3)	*ATXN3*	12–44	55–87	Ataxin 3	CAG•CTG	Exon

## Gain-of-Function Disorders

Expansion of CAG•CTG repeats is the hallmark of a number of different NRDs. The location of these sequences within the open reading frame of the corresponding disease genes varies and the biological pathways leading to phenotypes are different. However, CAG•CTG repeat expansion results in most cases in the production of toxic RNA and/or protein containing polyglutamine tracts. We have limited the scope of this review to include only the following CAG-related diseases: Huntington’s disease (HD), myotonic dystrophy type 1 and 2 (DM1 and DM2), and spinocerebellar ataxias 1, 2, and 3 (SCA 1, 2, and 3). Huntington’s disease is an autosomal dominant disorder characterized by progressive degeneration of nerve cells in the brain leading to movement, cognitive, and psychological impairment [7]. CAG expansion in exon 1 of the *Huntingtin* (*HTT*) gene results in toxic mutant (mutHTT) RNA and protein. Myotonic dystrophy is an autosomal dominant disorder characterized by progressive muscle weakness [8]. DM is the most common muscular dystrophy having an adulthood onset. It can be classified in two subtypes: type 1 (DM1) is caused by a CTG expansion in the 3′-untranslated region (UTR) of the myotonic dystrophy protein kinase (*DMPK*) gene, which encodes a myosin kinase. A milder phenotype was later identified in type 2 (DM2) and is caused by an unstable CCTG•CAGG repeat located at intron 1 of the nucleic acid–binding protein (*CNBP*) gene. SCA1, SCA2, and SCA3, also known as Machado–Joseph disease, are neurodegenerative disorders that belong to a large group of dominantly inherited spinocerebellar ataxias [9]. Expansion of CAG repeats in coding regions of the *Ataxin 1*, *2*, and *3* genes, respectively, is directly associated with the development of these diseases.

## Loss-of-Function Disorders

Friedreich’s ataxia (FRDA) and fragile X syndrome (FXS) are two of the most studied NRDs associated with loss-of-function mechanisms. FRDA is an autosomal recessive neurodegenerative disorder characterized mainly by ataxia, sensory loss, and motor dysfunction. Cardiomyopathy, diabetes, and scoliosis are other features associated with the disease. The majority of FRDA patients (98%) carries an expansion of a GAA•TTC repeat in the first intron of the *Frataxin* (*FXN*) gene on both alleles, whereas the rest (2%) has an expansion on one allele and a point mutation or deletion on the other [10]. The GAA•TTC expansion results in a deficiency of the corresponding Frataxin protein and therefore, current research focuses on therapeutic strategies that increase the amount of mRNA and/or protein to reach normal levels [11].

Fragile X syndrome (FXS) is an X-linked neurodevelopmental disorder caused by a CGG•CCG repeat expansion in the 5′-UTR of the *FMR1* gene [12, 13]*.* Expansion exceeding 200 repeats results in hypermethylation and silencing of *FMR1* and reduction in the level of the corresponding product, fragile X mental retardation protein 1 (FMRP) [14, 15]. The repeat expansion in the *FMR1* gene is a clear example of the complexity of NRDs being directly related to varying numbers of the corresponding nucleotide repeat (Table 1). Healthy individuals have ≤ 55 copies of the CGG•CCG repeat in the *FMR1* gene [16]. Males and females carrying 55–200 repeats (so-called premutated alleles) are at risk of developing fragile X-associated disorders, e.g., fragile X-associated tremor/ataxia syndrome (FXTAS) and premature ovarian failure (POF), respectively [17, 18]. Interestingly, expansion between 55 and 200 repeats results in an increased transcription of *FMR1* mRNA, but deficient translation to produce FMRP. On the other hand, transcriptional silencing, leading to FXS, is reached first when the expansion is > 200 repeats. In other words, ON treatment concepts in the case of the *FMR1* gene mutations cannot follow “straightforward” strategies, because expansion of the CGG•CCG repeat may lead to several different phenotypes.

## Chemical Modifications of Oligonucleotides

**Fig. 2:**
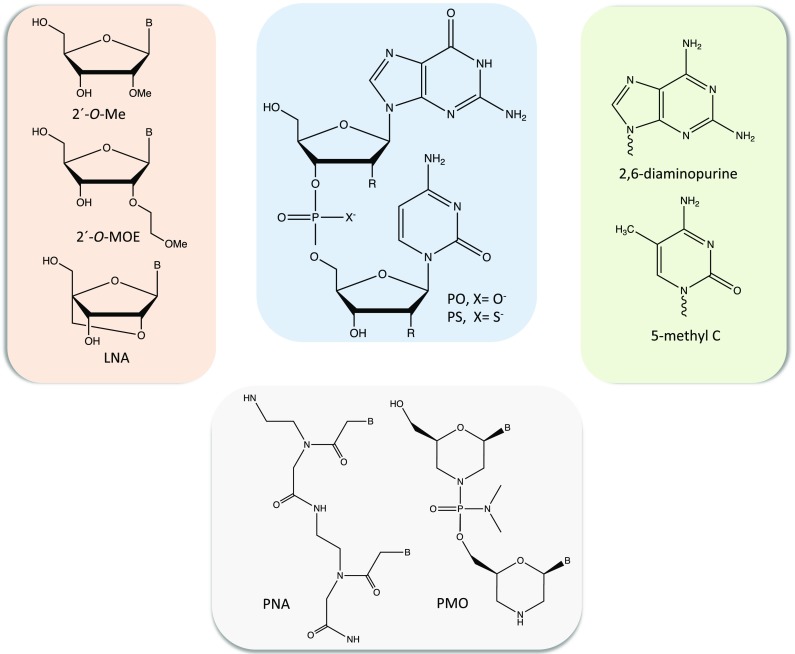
Chemical modifications of oligonucleotides. Chemical structure of a GC dinucleotide is shown (blue box) in which R = H for DNA and R = OH for RNA; PO = phosphodiester and PS = phosphorothioate. Examples of heterocyclic nucleobase (light green box) and sugar (light salmon box) modifications are shown. Two examples of sugar-phosphodiester backbone modifications are also presented (light gray box). Abbreviations: 2′-*O*-Me, 2′-*O*-methyl; 2′-*O*-MOE, 2′-*O*-methoxyethyl; B, heterocyclic nucleobase; 5-mehtyl C, 5-methylcytosine; LNA, locked nucleic acid; PNA, peptide nucleic acid and PMO, phosphorodiamidate morpholino oligomers

Oligonucleotides based on non-modified nucleic acids are readily degraded by endo- and exonucleases both in plasma and in the cell [19, 20]. A plethora of chemical modifications of ONs has been reported during the latest decades aiming to provide biologically active compounds with improved plasma half-life, cell uptake, stability, and bio-distribution in different tissues as well as enhanced target binding affinity and specificity [21]. ON modifications can be made at one or several of the following sites of a nucleic acid (Fig. 2): the heterocyclic nucleobase, the sugar moiety, the phosphodiester linkage, and/or the sugar-phosphate backbone. Here, we describe only a few ON chemical modifications (Fig. 2) that are relevant to the field of nucleotide repeat genes, thereby leaving out the remaining ON modifications (for more detailed reading, please see [21]).

One of the most examined nucleobase modifications in RNA-targeting ONs is 5-methyl cytosine (5-Me-C, Fig. 2). This substitution enhances duplex thermal stability, which is attributed to the stacking of the methyl group between the nucleobases in the major groove of the duplex [22]. Interestingly, the improved property is also valid for modified RNA guide strands in short interfering RNA (siRNA) and for ONs targeting double-strand DNA (dsDNA) [23]. Moreover, the 5-Me-C modification also prevents innate immune reactions from Toll-like receptor 9 [6, 24], which is expressed in the brain.

The 2′-position of the ribose sugar in RNA has an electron-withdrawing group, which results in a C3′-endo sugar pucker with a north conformation favorable for duplex formation; thereby, an RNA/RNA duplex is more stable than the corresponding DNA/DNA duplex. Several sugar modifications in antisense oligonucleotides (AONs) have, therefore, been developed to obtain an RNA-like structure. 2′-*O*-methyl (2′-*O*-Me) (Fig. 2) is a naturally occurring modification and is one of the most applied in several strategies, including in antisense [25, 26]. Another example of 2′-*O*-modification of ribose, 2′-*O*-methoxyethyl (2′-*O*-MOE), is shown in Fig. 2, which improves binding affinity to RNA and resistance to nucleases [21].

**Fig. 3:**
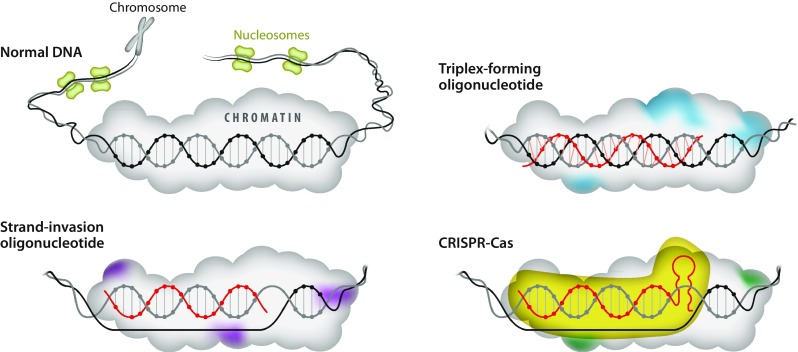
Genome targeting mechanisms. Chromosomal DNA and anti-gene mechanisms to interfere with the duplex through triplex-forming oligonucleotides, strand-invading oligonucleotides, or CRISPR-Cas, in which an optimized guide RNA can be made from a synthetic oligonucleotide. Colored chromatin represents modifications induced by the interference

Locked nucleic acid (LNA) was developed to further restrain the C3′-endo and north sugar conformation [27–29]. In LNA (Fig. 2), a methylene bridge links the 2′-*O* with the C4′ position resulting in excellent duplex-stabilizing properties. LNA-based ONs have been successfully used in siRNAs and AON gapmers, which work through RNA degradation and are described in more details in the following sections [30]. In addition, the LNA modification is used to improve binding of single-strand oligonucleotides (ssONs) and triplex-forming oligonucleotides (TFOs) to DNA to form duplex and triplex structures, respectively (Fig. 3) [31]. LNA analogs, or bridged nucleic acids (BNAs) such as 2′,4′-constrained ethyl (cEt) BNA [32], having constrained conformation, have also been synthesized and found to improve ON performance.

Phosphorothioate (PS) is a modification of the phosphodiester linkage in which one of the non-bridging oxygen atoms is replaced by a sulfur atom [33], as shown in Fig. 2. PS confers resistance towards exo- and endonucleases and extends the half-life of ONs in plasma, due to its protein binding ability [34]. However, the drawback of this later feature is non-specific protein binding, which may lead to undesired side effects such as immunostimulation, complement activation, and thromobocytopenia, in particular when PS is combined with additional ON modifications [35–37]. On the other hand, PS backbones can promote binding to proteins (such as La and NPM1), which increases AON nuclear localization [38].

An additional modification of the phosphodiester group is the introduction of a nitrogen atom at the 3′-position of the sugar moiety (ribose in RNA and deoxyribose in DNA) and hence substituting the 3′-OH [39]. N3′-phosphoramidate (NP) modification provides increased binding affinity of RNA and stability towards nucleases. However, NP is not recognized by RNase H and therefore cannot be part of the gap in gapmers (Fig. 2). Furthermore, the sugar and phosphodiester backbone can be replaced to generate two different classes of charge-neutral backbone: peptide nucleic acid (PNA) [40] and phosphorodiamidate morpholino oligomer (PMO) [41]. PNA and PMO (Fig. 2) are not degraded by nucleases, provide high binding affinity, and do not trigger RNase H. PNA is a nucleic acid mimic, which has a peptide-based backbone composed of *N*-(2-aminoethyl)glycine but is still able to bind to nucleic acids with higher affinity than non-modified DNA or RNA. Furthermore, PNA binds to dsDNA and forms double- and triple-strand structures (Fig. 3) through Watson-Crick, Hoogsteen, or reverse-Hoogsteen base pairing [42]. PNA [43] and LNA [44, 45] can invade dsDNA and form different PNA:DNA, and LNA:DNA, complexes, respectively (Fig. 3).

## The Choice of Target Sequence in the Context of Allele Selectivity

**Fig. 4:**
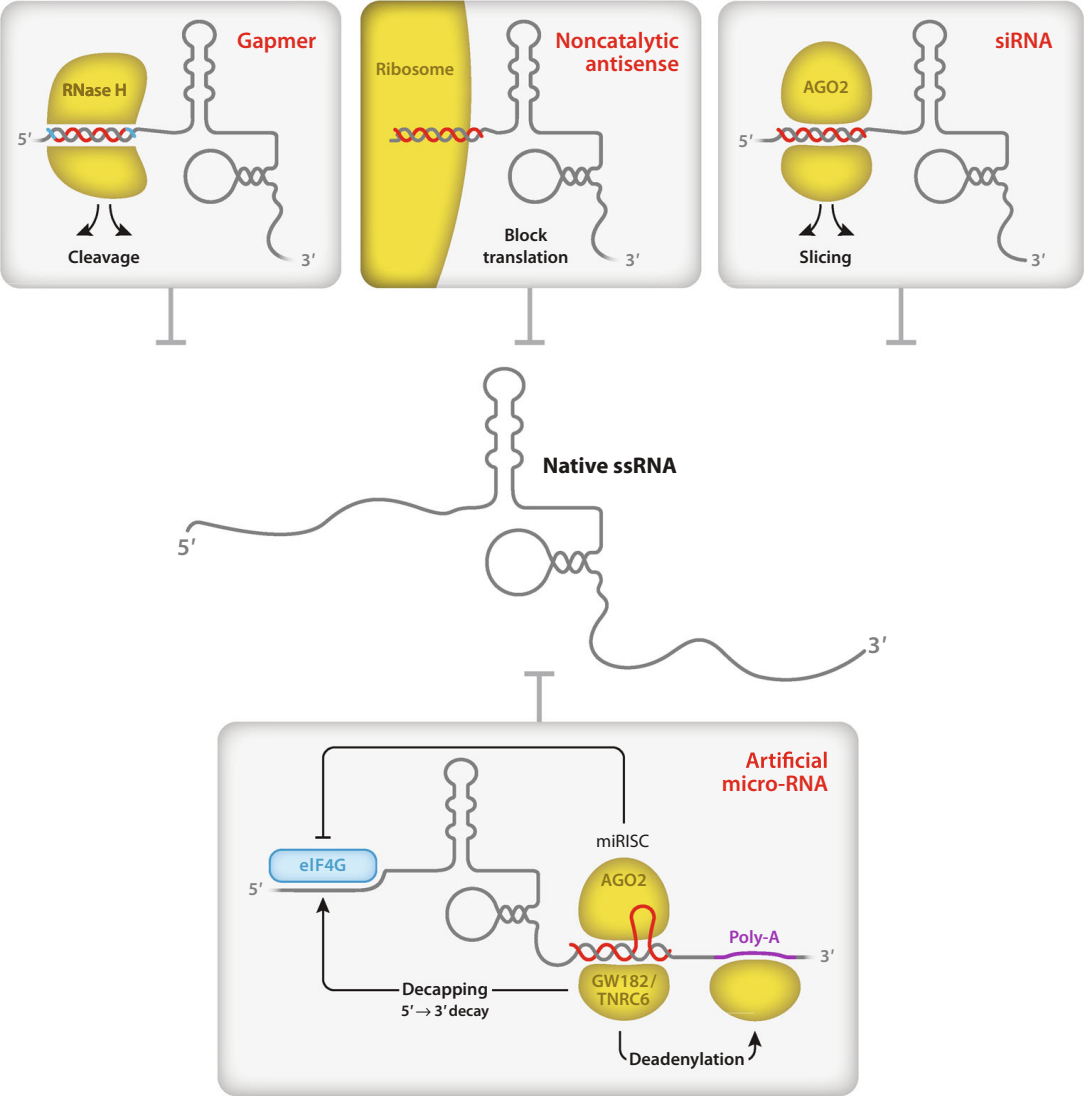
Oligonucleotide treatments directed against RNA and the corresponding mechanisms: RNA cleavage, block of translation, slicing and decapping and deadenylation. Center, target RNA; boxes show different approaches for RNA downregulation. Yellow indicates enzymatic processes. Upper left, RNase H cleaves DNA:RNA heteroduplexes and the gapmer contains a central DNA “gap” in red, surrounded by strongly hybridizing “wings” in blue. Lower panel, the eIF4G, eukaryotic translation initiation factor 4 G stabilizes capped mRNAs. GW182, glycine, tryptophan 182 kDa protein, alias TNRC6, trinucleotide repeat-containing gene 6A protein, interacts with the AGO2, argonaute protein, (also depicted in upper right panel) forming the micro-RNA-induced silencing complex (miRISC)

Already 40 years ago, Zamecnik and Stephenson first described the biological activity of synthetic AONs [46, 47], and such compounds remain the predominating ON therapeutic strategy for NRDs with dominant inheritance as depicted in Fig. 4 (reviewed in [6, 48]). In theory, for NRDs with this inheritance, the preferred principle would be to only target the disease allele and not the normal allele. There are, however, arguments against this view. Thus, in order to selectively target single alleles, they must differ. One option is that they are polymorphic outside the repeats. Such sequence polymorphisms vary among individuals. This means that many different therapeutic ONs need to be generated to obtain selectivity, and apart from constituting a technical challenge, this also leads to considerably more expensive therapies as compared to the use of a universal ON directed against a non-polymorphic target found essentially on all alleles. An alternative approach would be to direct the ON medicine against the repeat sequences in the mRNA provided that there would be differences in their secondary structure based on repeat length, which could discriminate the longer disease allele from the shorter, healthy one. There are indications from structure predictions that expanded repeats in transcripts may differ sufficiently for selective targeting of AONs [49]. However, favored binding of a longer repeat in RNA could only be expected based on the presence of significant discrepancies in both the length and conformation of the repeat sequences. It should also be emphasized that none of the AONs currently used has the capacity to completely silence expression of both alleles. This means that a certain level of intact mRNA and protein will always be generated.

To illustrate the complexity of the situation, we will provide an example, namely HD. It has been demonstrated that gene-targeted mice completely lacking the huntingtin protein die in utero, whereas the effect of heterozygosity may vary [50–52]. Mice with highly reduced expression showed perinatal mortality [53]. This clearly demonstrates that in this species, a certain level of huntingtin protein is needed during gestation. Whether the same is true in humans is not known, but none of the current therapeutic AON strategies would completely wipe out expression of *HTT*, and the resulting phenotype would therefore likely differ as compared to a gene knockout situation. In nonhuman primates (NHPs), it has been demonstrated that 45% downregulation of *HTT* following an RNAi approach did not induce any measurable abnormalities 6 weeks [54] or 6 months [55] after treatment. This does not exclude the possibility that more subtle changes still may occur, and again it is unknown whether these observations are translatable to humans, or if there are any differences among species.

Interestingly, when the effect of inactivating the *HTT* gene in mice was further analyzed, it was found that it was not the lack of expression in embryonic, but rather in extra-embryonic tissues that was the cause of the lethality [56]. If the same is true in humans, this observation is compatible with the use of nonallele-selective AONs as therapy after birth. Conversely, it was recently reported that conditional inactivation of the *HTT* gene in the adult mouse at 3, 6, or 9 months of age leads to progressive motor and behavioral decline, reduced life-span, and extensive neuropathology [57]. Collectively, although a cautious attitude is important, there is no definitive evidence suggesting that downregulation, i.e., not elimination, of huntingtin expression in the adult causes neuronal abnormalities. The same is also true for SCA1, because downregulation of ataxin 1 in macaques did not result in any detectable brain aberrations [58].

## RNA-Targeting Mechanisms in Gain-of-Function Disorders

A therapeutic aim in dominantly inherited NRDs is to lower toxicity by reducing the cellular mRNA content and as a consequence of that, also the protein levels. As depicted in Fig. 4, there are four major approaches, all of which have attracted interest from the scientific community. These are 1) single-stranded AONs (ssAONs), normally in the form of gapmers, which make use of RNase H for the degradation of the targeted RNA; 2) noncatalytic ssONs, which block translation of mRNA to protein; 3) micro-RNA-mediated silencing and 4) predominantly double-stranded AONs (dsAONs) used in siRNA-mediated downregulation. Gapmers contain a central “gap” of ssDNA, which upon hybridization to RNA permits degradation of the RNA by the endogenous RNase H enzyme. The gap is on both sides surrounded by a short stretch of modified nucleotides, which normally are resistant to degradation and show strong binding. Gapmer ssAONs is currently the prevailing ON approach for treatment of dominantly inherited diseases (Fig. 4) with HD being the most extensively studied among the NRDs, presumably owing to its prevalence and unique, characteristic phenotype.

Rather than reviewing all studies related to RNA-targeting approaches, we have chosen to identify some of the earliest reports and to concentrate on certain aspects of more general interest taking into account the universal considerations on how to apply therapeutic ONs.

## Single-Strand Antisense Oligonucleotides

The first antisense approach, using 18mer PS AONs directed against *HTT* transcripts and injected into the striatum of mice, was reported in 1997, but failed to induce downregulation [59]. Yen et al. [60] used a catalytic DNAzyme, which reduced *HTT* levels in cells, whereas Nelleman et al. in 2000 [61] were the first to report on the successful use of a phosphorothioate (Fig. 2) AON to reduce huntingtin protein levels in cells (Fig. 4). Transient infusion of nonallele-selective gapmer AONs into the cerebrospinal fluid in HD mouse models delays disease progression and also mediates a sustained reversal of disease phenotype, which persists longer than the huntingtin knockdown [62]. Using this strategy, infusion in NHPs also effectively lowered huntingtin in many brain regions affected by HD pathology [62]. Such strategies have also been attempted in other disorders with polyglutamine expansion, such as in spinocerebellar ataxia type 3, SCA3 [49, 63], and in SCA1 [64]. Also the *DMPK* transcript, defective in DM1 has been targeted using AONs [65]. Moreover, two ONs, a 2′-*O-*methoxyethyl/2′,4′-constrained 2′-*O*-ethyl (2′-MOE/cEt) and a 2′-MOE gapmer AONs (Figs. 2 and 3) were used as treatment in a mouse model of Spinal and bulbar muscular atrophy (SBMA). The authors reported that a single intracerebroventricular administration of the antisense ONs in the pre-symptomatic phase suppressed mutant gene expression in the CNS and delayed the onset and progression of motor dysfunction [66].

In addition, splice switching has been studied in SCA3 mice with the aim of skipping the exon encoding the repeat [67]. The basic concept in splice switching is to use ON binding to the pre-mRNA to alter the inclusion of exons, a strategy, which has been studied extensively in Duchenne muscular dystrophy (DMD) with the aim of restoring the reading frame [6]. For most proteins, exon skipping leads to abolished activity, whereas for dystrophin, the activity is only slightly reduced.

## Disease Allele–Selective Approaches

Owing to that in autosomal dominant disorders there is one mutated and one healthy allele, it may be possible to direct the therapy only to the affected allele. As discussed in the previous section, this would be an elegant solution, but when targeting single nucleotide polymorphisms (SNPs), this is normally achieved at the expense of more individualized and costly treatments. Suitable candidate SNPs have been identified in, e.g., the *HTT* gene [68].

Allele-selective HTT gapmers, in which a central DNA gap is surrounded by 2′-*O*-MOE wings, for enhanced hybridization and stability, were previously developed [69] and subsequently tested both in mice and in NHPs demonstrating good activity. In NHPs, suppression of HTT was observed throughout the cortex and limbic structures [70], and by rational design, it is possible to obtain AONs highly selective for the *HTT* disease allele [71].

## Allele-Selective Targeting Using Repeat-Directed Antisense Oligonucleotides

Interestingly, by targeting the repeats in the transcript, it has been possible to obtain allele selectivity in both SCA3 and in HD. In these studies of cultured cells, several different nucleic acid chemistries were used in the single-strand AONs, including substitution using LNA, or PNA [49]. Prediction of the mRNA secondary structure carrying repeats of different lengths suggested that allele-selective targeting could be achieved by the use of nonpolymorphic oligomers. Apart from secondary structures yielding selectivity, the increased length of the repeat in the disease allele per se would also enhance targeting simply because additional cognate sequences would be available for hybridization.

## Allele-Selective siRNAs

Apart from using allele-selective gapmer AONs, efforts have also been made to apply allele-selective siRNAs (Fig. 4). It has been reported that the majority of SNPs in the *HTT* gene are intronic [72]; however, siRNAs are active only in the cytoplasm, i.e., after the introns have been removed from the pre-mRNA. In contrast, gapmers function both in the nucleus and in the cytoplasm [73], and thereby enable more options for the targeting of SNPs. In a recent report [74], it was demonstrated that about half of the *HTT* mRNA in neuronal cells is located in the nucleus. This further argues in favor of therapeutic strategies efficiently targeting nuclear-resident nucleic acids. Most of the RNAi-based studies to date have been based on viral transfer of the RNAi construct [75–77], but there are also attempts to develop chemically modified ONs for this purpose [78].

## Construction of Micro-RNA Mimics

A novel approach for the treatment of HD is based on targeting of the 3′ end of *HTT* mRNA (Fig. 4), a region frequently used in endogenous micro-RNA regulation. Through the development of an adeno-associated virus encoding artificial micro-RNAs, it has been possible to harness the cellular machinery for degradation of *HTT* mRNA in both rats [79] and in minipigs [80]. The US Food and Drug Administration granted orphan drug designation for this therapy, named AMT-130, in Huntington’s disease in 2017 and in January 2018, AMT-130 received an Orphan Medicinal Product Designation (OMPD) from the European Medicines Agency for the same indication. Although this development is based on therapeutic viruses, the same approach could be applied by the use of ON-based micro-RNA mimics. Such attempts have already been made and entered into clinical trials in the field of tumor treatment in which RNA species, that are believed to have transforming activity, have been targeted, as reviewed in Smith and Zain [6].

## Clinical Trials

In 2018, the first results from a phase I/II study using a 20mer 2′-MOE gapmer directed against *HTT* transcripts were announced (Ionis Pharm. 2018. http://ir.ionispharma.com/node/23401/pdf). The Roche/Ionis gapmer directed against *HTT* RNA, designated RG6042, also known as IONIS-HTTRx, received PRIME designation by the European Medicines Agency (EMA) in August 2018, primarily based on the data from an exploratory phase I/IIa trial demonstrating significant reduction in mutated huntingtin in the cerebrospinal fluid of adult patients treated for 3 months. The levels of the defective protein continued to decline in the majority of treated patients and in January 2019 it was announced that the first patient has been enrolled in a phase III study of RG6042.

## Genome and Transcript Editing

During the twenty-first century, there have been major developments in the field of genome editing. For decades, this was considered as science fiction, but the availability of increasingly efficient methods has changed the situation completely. Thus, tools such as zinc finger nucleases (ZFNs), transcription activator–like effector nucleases (TALENS), and the clustered regularly interspaced short palindromic repeat (CRISPR)–associated protein system are available and the current development is unprecedented [81, 82]. We will only very briefly discuss the CRISPR-Cas technology, which is schematically depicted in Fig. 3. The CRISPR-Cas complex is composed of an enzymatic component and a guide RNA (gRNA). Improvement of the catalytic activity and specificity has been made using gRNAs carrying synthetic, chemically modified nucleotides [6].

Studies in mice suggest that nonallele-selective CRISPR/Cas9-mediated gene editing by deletion of the *HTT* repeats could be used to permanently eliminate polyglutamine expansion-induced neuronal toxicity in the adult mouse brain [83, 84]. Excision of the CAG tract from the *HTT* gene by Cas9 nickases was also recently reported [85]. Furthermore, also for other NRDs, such as the fragile X syndrome, Friedreich’s ataxia, and SCA2 and SCA3, genome editing has been tested [86–89]. In DM1 and DM2, the reported approach was based on the use of deactivated editing enzymes, which efficiently reduced transcription after systemic delivery of dCas9/gRNA by an adeno-associated virus vector [90]. Editing occurs preferentially in dividing cells, and it was recently demonstrated that Cas9-mediated cleavage of DNA is quite weak when nucleosomes are present, whereas the activity of ZFNs was less affected [91]. Engineered systems, which target the defective RNA, have also been described for HD, DM1, and DM2 [92].

Endonucleases may have different molecular weights; nevertheless, their size remains a hurdle for uptake when many cells in the brain need to be targeted, but it is evident that curative, clinical genome editing for NRDs may become possible in the future. Delivery of the editing enzyme, in complex with the synthetic chemically modified gRNA, and virus-mediated transfer are possible scenarios for such therapies.

## Nucleic Acid–Based Approaches in Loss-of-Function Disorders

Repeat expansion can interfere with the regulation of gene transcription in several different ways, including bidirectional and antisense transcription, formation of RNA:DNA hybrids [93], and non-B-DNA structures [2]. In the case in which the repeat expansion leads to reduced expression of mRNA and protein, “classical” AON targeting of RNA (as described in a previous section) is clearly not an option. In FRDA, expansion of the GAA•TTC repeats is directly associated with transcription downregulation, which results in reduced levels of frataxin mRNA and protein [94, 95]. Inhibition of Pol II in FRDA is also correlated with repressive chromatin modifications, and gene silencing of FRDA has been described. A novel approach to circumvent the frataxin deficiency is to directly deliver the corresponding mRNA *in vivo*. Recently, transfer of human *FXN* mRNA was first examined in 293-T cells using lipofectamine transfection and mature functional FXN protein was detected. More important, lipid encapsulated nanoparticles of *FXN* mRNA were subsequently delivered by intrathecal injection in adult mice and human FXN protein was measured in the dorsal root ganglia [96]. Another strategy is to direct chemically modified ONs to the repeat region of *FXN* pre-mRNA to avoid its engagement in forming RNA:DNA hybrids, which has been suggested as one of several possible mechanisms leading to the deficiency of frataxin mRNA and protein [97, 98].

## RNA Targeting

To affect gene expression in loss-of-function diseases using ONs directed against RNA, one has to consider several aspects, which differ from targeting toxic mRNA in gain-of-function diseases. For example, in Friedreich’s ataxia, the expanded GAA•TTC repeats are located in an intron and therefore, ONs directed to the repeat region of the transcript should 1) target the pre-mRNA and exert their activity in the nucleus and 2) lead to an activation, rather than an inhibition, of gene expression.

Double-strand RNA (dsRNA) has been examined for activating *FXN* gene expression in Friedreich’s ataxia patient–derived fibroblasts [97]. To this end, dsRNA targeting the GAA•TTC repeat in the RNA enabled transcription elevation, which has been attributed to an RNAi de-repression mechanism. In this study, the authors reported that Argonaute2 (Ago2) binding of the transcript is necessary, but without the engagement of an Ago2-mediated cleavage. Moreover, an antisense LNA-based ON targeting the pre-mRNA at the repeat region was shown in the same study to cause activation of *FXN* gene expression. Although the gene-activating molecular mechanism of the AON has not been fully examined, one possible scenario is that the ON causes release of the pre-mRNA from a DNA:RNA hybrid (sometimes referred to as R-loop), which has been proposed to form at GAA and CGG repeats [99, 100].

Fragile X-associated tremor ataxia syndrome (FXTAS) is caused by a CGG repeat expansion in the *FMR1* gene [16]. However, and as mentioned in the previous section, CGG•CCG expansions of > 200 repeats are also associated with fragile X syndrome. The CGG repeat is located in the 5′-untranslated region and pathological repeats of 55–200 produces toxic RNA. AONs carrying 2′-*O*-Me-PS have been examined in model cell lines with the aim to invade the structured RNA formed at the 5′-UTR of the *FMR1* gene [101]. Nevertheless, this strategy may not be optimal, as inactivation of the *FMR1* mRNA may aggravate disease because fragile X syndrome is caused by loss of FMRP. Instead, low-molecular weight small molecules have been screened for their ability to recognize and bind the structured transcript.

## Natural Antisense Transcripts and Oligonucleotide Treatment

Natural antisense transcripts (NATs), which are complementary to the corresponding mRNA, are heterogeneous and prevalent and often accumulate in the nucleus [102, 103]. They likely have regulatory functions [102], but their rather modest expression level [104] has made it difficult to clearly establish their role. The opposite directional transcription of antisense and sense RNAs suggests that they might be part of self-regulatory circuits allowing genes to control their own expression.

NATs are associated with the *DMPK* [105], *FXN* [106], *HTT* [107], and *SCA2* [108] genes. The expanded disease allele in congenital DM1 is associated with loss of CCCTC-binding factor (CTCF) binding, spread of heterochromatin, and regional CpG methylation. In Friedreich’s ataxia, *FXN* antisense transcription and depletion of the chromatin insulator protein CTCF are also associated with epigenetic silencing [106, 109]. In a recent report, a *FXN* NAT was also found to exert an effect when expressed in *trans* [110]. In the *HTT* gene, a NAT named *HTTAS*, encompasses the *HTT* locus containing the repeat tract [107]. It is 5′-capped, poly (A) tailed and contains three alternatively spliced exons expressed in multiple tissue types and throughout the brain. Repeat expansion seems to reduce the efficiency of the corresponding promoter. Over-expression of one splice form specifically reduces endogenous *HTT* transcript levels. Collectively, these findings support the idea that NATs may regulate the expression of many NRD genes.

Owing to that the physiological role of the NATs to a great extent is unknown, their potential role as drug targets also remains elusive. It is possible to influence the levels of the NRD-associated NATs by, e.g., AONs, but whether this will have any beneficial clinical effect is not known. To this end, it has been demonstrated that in SCA2, a NAT exerts toxic functions and it has been proposed that therapeutics against these transcripts may have favorable effects [108].

The frequent existence of NATs also means that whenever the effects of ON-based treatments for NRDs are evaluated, such transcripts need to be taken into consideration. Depending on their sequence, and which genomic region they correspond to, AONs directed against the regular sense transcript may resemble parts of a NAT and, conversely, an AON complementary to the NAT may be identical to a stretch of the regular sense transcript. In a similar way, ONs directed towards genomic DNA (anti-gene ONs) may target, or be similar in sequence, to the NAT depending on their composition. In the experiments recently conducted in our laboratory using anti-gene ONs directed against the *HTT* gene, the anti-gene ONs target the template strand and are hence identical in sequence to a stretch in the *HTT* mRNA [111]. An effect on the NAT is unlikely, because that presumably would result in the upregulation of the sense *HTT* mRNA, whereas we observed the opposite. This is also in line with the demonstration that an siRNA directed against the *HTT* NAT causes enhanced *HTT* mRNA expression [107].

## Structural Properties and Genomic Instability of Repeats

**Fig. 5:**
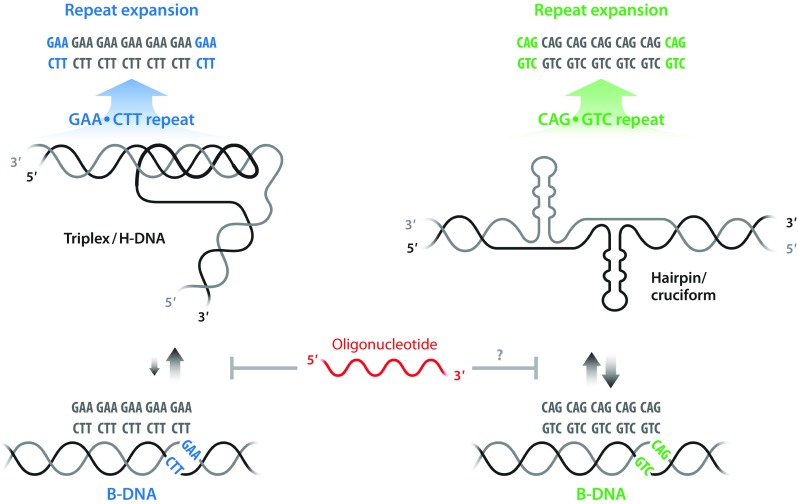
Oligonucleotide targeting of non-B-DNA structures at expanded repeat regions. Schematic representation of genomic DNA carrying repeat expansions, in which the presence of GAA•TTC repeats promotes the conversion of B-DNA to a pyrimidine motif (YRY) triplex/H-DNA structure in Friedreich’s ataxia (left) and CAG•CTG repeat expansions, such as in Huntington’s disease, adopt a hairpin/cruciform conformation (right). The gray arrows indicate the direction and strength of the equilibrium between B- and non-B-DNA structures, indicating higher propensity to form a stable triplex/H-DNA in GAA•TTC repeats (left) compared to the conformations formed in repeats of CAG•CTG (right). The red oligonucleotide interferes with formation of non-B-DNA structures, which are implicated in the repeat instability leading to expansions (top panel)

The most common DNA structure in the human genome is the right-handed helix known as B-DNA. However, repeat sequences within DNA can form non-canonical conformations, which are collectively called non-B-DNA. These structures are formed at different repeat sequences; e.g., CAG•CTG repeats can adopt a hairpin or cruciform conformation (Fig. 5), whereas polypurine•polypyrimidine mirror repeats (such as GAA•TTC repeats) form an inter- or intramolecular triple-helix (triplex), also known as H-DNA (Fig. 5). G-rich sequences can form G-quadruplex (G4) structures consisting of π–π stacking of planar G-tetrads. These conformations are implicated in NRDs even if this has not been equally substantiated for all structures [2, 5].

An important parameter for the expansion rate is whether a tandem repeat sequence is uninterrupted or not. It has been demonstrated that interruptions profoundly reduce the expansion rate, and the length of the uninterrupted repeat determines the stability [112]. Here, it was shown that the composition and the length of the repeat determine the propensity to form secondary structures, such as hairpins. The structure and the dynamics of both DNA and RNA duplexes of CAG, GAC, CCG, and GGC trinucleotide repeats have been studied by molecular modeling revealing that A-A non-canonical pairs form high-anti conformations in DNA [113] and that mismatches in C-rich hairpin stems are weakly bonded and may flip out forming “e-motifs” [114, 115]. Although these studies provide interesting insights, the detailed dynamics of these processes inside the nucleus of neuronal cells remains to be elucidated.

It is believed that changes in the repeat number in normal cells mainly occur during replication, transcription, or under DNA repair (reviewed in [3–5]). In addition, they may occur when translocations take place. It has been demonstrated that extended hairpins have long lifetimes, even in the presence of their complementary strands, and inhibit duplex reannealing at a slippage site [116]. For repeated CG-containing sequences, RNA:DNA hybrids can form at the expanded, abnormal, CGG repeat regions, as shown in the *FMR1* gene [117]. Apart from introducing structural changes, this also may result in silencing of this gene. Chromatin domain boundaries were recently reported to co-localize with short tandem repeats [118], and in plants, it was found that an intronic GAA•TTC repeat induces accumulation of siRNAs and repressive histone marks, causing epigenetic silencing [119].

Owing to that the main expansion in NRDs occurs in disease-specific subsets of neuronal cells in the adult [120, 121], and that these cells only rarely divide, transcription and DNA repair are considered as the most important contributors for the observed plasticity. Apart from that transcription causes strand separation, it also yields negative supercoiling, which further enhances the stability of non-B-DNA structures [122, 123]. The isolation of a large number of mutants prone to GAA•TTC fragility and large-scale expansions in yeast suggest that transcription initiation in nondividing cells is crucial for genome instability [124]. For Huntington’s disease, a catalog of genetic components that modify the clinical onset of disease was recently compiled [125]. It included the MutL Homolog 1 (*MLH1*) gene, substantiating a role for DNA repair and this gene, among several others, was also reported in what is referred to as a “repeat expansion DNA damage response (REDD) pathway” that acts to prevent repeat expansions in the genome [126]. Related repair proteins were also found in other studies [127]. *Cis*- and *trans*-modifiers of repeat expansions were recently reviewed [5]. Collectively, this shows that there are numerous cellular components, which influence the propensity to expand tandem repeats, although considerably less is known about what ignites these processes.

## Oligonucleotide Targeting of Non-B-DNA Structures

The ability of expanded nucleotide repeat sequences to form alternative DNA structures provides an additional possibility to target the mutated gene allele and this is being investigated in preclinical models. Modified ONs can then be targeted to interact with the repeats at the site of non-B-DNA structure. A prime example is expanded GAA•TTC repeats in the *Frataxin* gene in Friedreich’s ataxia, which can form an intramolecular triplex (H-DNA). There are few examples in which this concept has been examined using repeat-specific oligomers or modified ONs. One is the use of synthetic polyamides, initially developed to recognize dsDNA and form a triplex structure (Fig. 3); however, their binding was shown to rather occur through recognition of the dsDNA minor groove [128]. Polyamides have been previously examined aiming to modulate gene expression in different cell models, both through transcription inhibition and activation. In FRDA lymphoid cell lines, GAA-binding polyamides showed moderate enhancement of the levels of frataxin mRNA and protein [129]. The effect was attributed to alteration of the DNA conformation or to chromatin opening through displacement of repressor proteins causing a reversal of inactive heterochromatin [130]. More recently, it was demonstrated that GAA-binding polyamides rescued replication fork stalling in FRDA-induced pluripotent stem (iPS) cells [131].

An approach that we have introduced is to take advantage of modified ONs, such as LNA/DNA mixmer ONs or PNA oligomers, which have the ability to invade dsDNA and form stable complexes. A detailed molecular analysis of the DNA structure(s) formed at FRDA GAA•TTC repeats has been carried out in our laboratory and we were able to confirm formation of a pyrimidine motif (YRY) H-DNA [132, 133], as shown in Fig. 5. Furthermore, we found that sequence-specific binding using repeat-specific PNA oligomers resulted in complete disruption of the triplex formed and LNA ONs showed similar results (Fig. 5) [42]. These findings are currently employed in FRDA patient cell lines to upregulate *FXN* transcription and increase the levels of mRNA and protein (manuscript in preparation). Nevertheless, these findings need to be substantiated in preclinical models.

Following a related DNA targeting concept, we have recently reported that LNA/DNA mixmer ON binding of CAG repeats in the *HTT* gene in primary patient cell lines resulted in efficient downregulation of transcription [111]. The ON used was directed against the template strand and we found no evidence of hybridization with *HTT* transcripts. Expanded CAG repeats can form a hairpin/cruciform structure, which we believe facilitates strand invasion and binding of the single-strand LNA ONs to a DNA strand (Fig. 5).

## Conclusion

The recent development of nucleic acid–based therapeutic strategies is deep-rooted in the long and extensive research in the fields of ON chemistry and biophysics, reliable biological models, imaging and delivery, to mention few. Nucleotide repeat diseases are biologically diverse, yet they share common features caused by genetic instability, mainly in the form of repeat expansion. The various ON mechanisms of action, targeting RNA, editing the genome, or interfering with the repeat DNA structure, have obvious potentials but each approach can prove to be suitable for certain but not all diseases. Targeting the repeat sequences at the DNA level and thereby preventing instability would be highly useful and is part of our ongoing research. Many approaches are under study and we are witnessing a promising scientific era that could enable important medical advancement for the treatment of several, if not all, nucleotide repeat disorders.

## Electronic supplementary material

ESM 1 (PDF 552 kb)
